# Glycolate from microalgae: an efficient carbon source for biotechnological applications

**DOI:** 10.1111/pbi.13078

**Published:** 2019-02-04

**Authors:** Anja Taubert, Torsten Jakob, Christian Wilhelm

**Affiliations:** ^1^ Department of Plant Physiology Institute of Biology University of Leipzig Leipzig Germany

**Keywords:** *Chlamydomonas*, photorespiration, glycolate

## Abstract

Glycolate is produced in autotrophic cells under high temperatures and C_i_‐limitation via oxygenation of ribulose‐1,5‐bisphosphate. In unicellular algae, glycolate is lost via excretion or metabolized via the C_2_ cycle by consuming reductants, ATP and CO_2_ emission (photorespiration). Therefore, photorespiration is an inhibitory process for biomass production. However, cells can be manipulated in a way that they become glycolate‐producing ‘cell factories’, when the ratio carboxylation/oxygenation is 2. If under these conditions the C_2_ cycle is blocked, glycolate excretion becomes the only pathway of photosynthetic carbon flow. The study aims to proof the biotechnological applicability of algal‐based glycolate excretion as a new biotechnological platform. It is shown that cells of *Chlamydomonas* can be cultivated under specific conditions to establish a constant and long‐term stable glycolate excretion during the light phase. The cultures achieved a high efficiency of 82% of assimilated carbon transferred into glycolate biosynthesis without losses of function in cell vitality. Moreover, the glycolate accumulation in the medium is high enough to be directly used for microbial fermentation but does not show toxic effects to the glycolate‐producing cells.

## Introduction

Photosynthesis is the most efficient process to convert CO_2_ from the atmosphere into organic matter by the help of water and sunlight. However, the efficiency becomes lower when photosynthesis drives the formation of biomass, since biomass formation is a slow and complex regulated process and finally limits energy conversion efficiency (Wilhelm and Jakob, 2011; Wilhelm and Selmar, 2011). Many attempts have been made to improve the efficiency of biomass formation in higher plants (Zhu *et al*., 2010) and in algae (Beer *et al*., 2009). Microalgae have been widely discussed as potential source for carbon‐based products like lipids (reviewed by Ho *et al*., 2014), carbohydrates (for instance starch; reviewed by Zachleder and Brányiková, 2014), proteins (reviewed by Bleakley and Hayes, 2017) or high valuable products (e.g. pigments, vitamins or antioxidants; for review see Chew *et al*., 2017). However, the high potential in algal biotechnology is still not fully exploited because of several reasons. Although the technology of photobioreactors has been significantly improved, the energy input for mixing, nutrient supplement, harvesting and refinement are still too high to obtain a significantly improved greenhouse emission balance compared to fossil fuels (Weinberg *et al*., 2012). This is especially the case when bioenergy, e.g. biofuels, are the target substance (Dassey *et al*., 2014; Quinn *et al*., 2014). Different approaches have been tested to overcome these limitations. One strategy is ‘milking’ the cells by in situ extraction of the target substance (Hejazi and Wijffels, 2004; Racheva *et al*., 2018). Although this idea was published more than 10 years ago, the technological readiness of this approach is still on the level of research and milking by in situ extraction still separates algal growth and harvesting in two different production phases and a continuous bioproduction is not achieved and thus, the energy conversion efficiency is reduced.

Based on these limitations, Günther *et al*. (2012) have proposed a new approach to use glycolate excretion as a natural process of C‐milking from the cells. It has been shown that glycolate can be directly used for anaerobic fermentation to produce methane. This approach is different from conventional biomass‐based biofuel production as it possesses a number of intrinsic advantages. The biosynthesis of glycolate drains the assimilated carbon from the biomass forming cellular pathways and involves far less reaction steps than the production of cellular biomass. In this way, the metabolic costs of the cells are drastically reduced. The active excretion of glycolate by the algal cells does further avoid harvesting and refinement processes that are necessary in biomass‐based approaches. Finally, the supplement with nutrients (except from CO_2_) will be drastically reduced since glycolate is a pure hydrocarbon molecule and it is aimed to drain photosynthetic energy and assimilated carbon from biomass production to glycolate production as efficiently as possible. However, this elegant approach can be applied successfully only if the following questions can be answered positively.

(1) Glycolate toxicity

Glycolate is produced in the chloroplast by the photorespiratory process where the enzyme Ribulose‐bisphosphate‐carboxylase/oxygenase (RubisCO) uses molecular oxygen instead of CO_2_ as a substrate. However, glycolate is also a toxic metabolite for the C‐assimilation process (Dellero *et al*., 2016). Therefore, glycolate has to be exported immediately from the chloroplast and metabolized to maintain cell viability in photosynthetic organisms. This is achieved by the activity of the phosphoglycolate phosphatase which dephosporylates 2P‐gycolate to free glycolate which is then the substrate of a membrane‐bound glycolate transporter (Pick *et al*., 2013). Glycolate is then oxidized to glyoxylate and further metabolized in the C_2_ cycle (Stabenau and Winkler, 2005). Rademacher *et al*. (2016) could show that red algal cells with reduced glycolate oxidase capacity cannot survive under ambient CO_2_ because of glycolate accumulation in the cells. In higher plants, the inactivation of the C_2_‐cycle leads to changes in the carbon allocation pattern and to an earlier senescence. However, in some green algae, like *Chlamydomonas*, the inactivation of the C_2_‐cycle does not induce glycolate accumulation inside the cells because of an active excretion of glycolate (Garbayo *et al*., 2005; Vílchez *et al*., 1991). For a biotechnological use of glycolate excretion by algal cells, a high glycolate concentration in the surrounding medium is required (see below). It is, however, completely unknown whether such high extracellular glycolate concentrations do have a negative impact on photosynthetic performance and cell vitality.

(2) Regulation of carbon assimilation and glycolate excretion.

The photorespiratory process loses cellular carbon by the C_2_ cycle or by the direct excretion of glycolate which could lead to cell death, finally. Therefore, to use glycolate excretion as biotechnological process, the cells must be enabled to resupply the photorespiratory carbon loss in parallel to glycolate excretion. This requires a continuous switching of RubisCO between the carboxylation and the oxygenation reaction. Normally, it is very difficult to manipulate specific C‐fluxes in the cell. However, in this case the kinetic features of the RubisCO are a big help. If the carbon concentrating mechanisms are completely inactivated (see below), the ratio O_2_/CO_2_ defines the mode of action of the RubisCO. This means in practice that a low ratio O_2_/CO_2_ (e.g. CO_2_‐enriched ambient air) promotes the carboxylation reaction, whereas a high ratio O_2_/CO_2_ (e.g. O_2_‐enriched ambient air) switches RubisCO into the oxygenation mode. Therefore, the gas composition has to be adjusted to a specific O_2_/CO_2_ level that ideally allows two carboxylation reactions per one oxygenation reaction per time. In this way, maximized glycolate production without draining of structural carbon from the cells but also without channelling of assimilated carbon into new biomass production could be achieved. For an efficient glycolate excretion not only the inactivation of all carbon concentrating mechanisms (CCM; Hagemann *et al*., 2016) but also the inhibition of glycolate metabolization by the C_2_ cycle (e.g. by inhibition of the glycolate dehydrogenase; GlyDH) is required. It was shown that CCMs and GlyDH can be blocked by genetic manipulation (Van *et al*., 2001) or by the addition of specific inhibitors (Zuo *et al*., 2014). We found that the inhibitor 6‐Ethoxy‐2‐benzothiazolesulfonamide (EZA) blocks both, the CCMs and the GlyDH, very efficiently. However, it is completely unknown how long green algal cells can survive without any cell division and what happens with the photosynthetic activity under a permanent high photorespiratory burden.

(3) Temperature dependence of photorespiration

The photorespiration is strongly dependent on the temperature (Aboelmy and Peterhansel, 2014). If photorespiration is used as a physiological mechanism for biotechnological means it must be shown that glycolate excretion is a robust process also in oscillating light and temperature regimes as experienced by algal cells in a bioreactor under ambient outdoor conditions.

(4) Sufficient glycolate concentration in the medium

Finally, for biotechnological reasons the glycolate concentration in the medium should yield a level high enough to be directly used for feeding the fermentation process. Otherwise the glycolate‐enriched algal medium cannot be used for fermentation without an additional concentration step which would be an important disadvantage of the complete process. This means, however, that the cells have to produce and excrete glycolate over long‐term period. Currently, it is not known whether it is possible to keep algal cells under photorespiratory conditions for the required time period of several weeks.

In the present study, cells of the green alga *Chlamydomonas reinhardtii* were cultivated under specific culture conditions to answer the questions raised above and with the aim to proof the biotechnological applicability of algal‐based glycolate excretion as a new biotechnological platform.

## Results and discussion

According to the different physiological state of the cells the results of the experimental approach were separated into three phases: (i) the biomass‐producing phase, (ii) the transition phase from biomass‐ to glycolate‐producing conditions and (iii) the long‐term glycolate‐producing phase.

### Biomass production conditions

**Figure 1:**
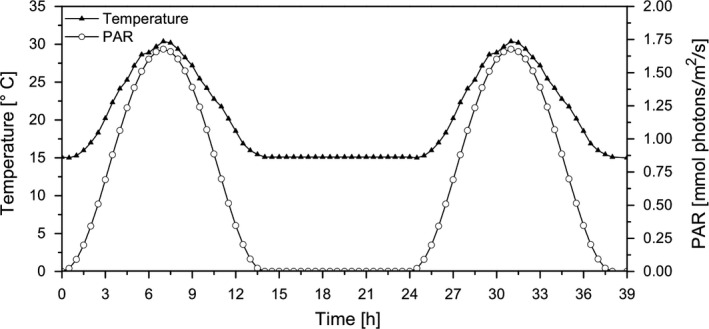
Daily course of temperature and light gradients during a 2‐day period. Temperature changed from 15 °C in the dark phase to 30 °C at midday maximum. Irradiance was applied with a sinusoidal shape and a maximum intensity of 1700 μmol photons/m^2^/s.

The biomass production phase served as reference condition and allowed to compare the biomass production potential with the glycolate production rate, subsequently. A photobioreactor setup with an illumination and temperature gradient close to natural conditions during a summer day was applied (Figure 1). After 5 days of continuous cultivation of *C. reinhardtii*, the cells were acclimated to these conditions which was reflected in a very constant chlorophyll *a* concentration and cell number (Table 1). The outdoor‐like growth conditions induced a high growth rate with a daily primary production of 3.5 mm carbon per mg Chl*a*.

**Table 1:** Cultivation and physiological parameters of *C. reinhardtii* under biomass‐ and glycolate‐producing conditions

	Biomass production		Glycolate production	
	Mean value	Standard deviation	Mean value	Standard deviation
Chl*a* [mg/L]	2.01	0.16	2.75	0.50
Cell number [10^9^/L]	2.35	0.20	3.27	0.50
Growth µ/[day]	2.22	0.12	0	0
Dry weight [mg/(mg Chl*a*)]	37.61	2.30	n.d.	–
C_BM_ [mmol C/(mg Chl*a*)/day]	3.50	–	n.d.	–
Min. daily prod_gly_ [mm/day]	n.d.	–	1.14	0.06
Max. daily prod_gly_ [mm/day]	n.d.	–	2.62	0.06
Max. c_gly_ [mm]	n.d.	–	40.63	0.81
Min. C_expect_: C_achieved_ [%]			36	
Max. C_expect_ : C_achieved_ [%]			82	

Chl*a*, Chl*a* concentration in algal suspension; µ, growth rate; C_BM_, carbon‐based biomass production rate; Prod_gly_, Glycolate production rate; c_gly_, Glycolate concentration in culture medium; C_expected_, Expected carbon assimilation rate; C_achieved_, Carbon assimilation rate measured as excreted glycolate.

**Figure 2:**
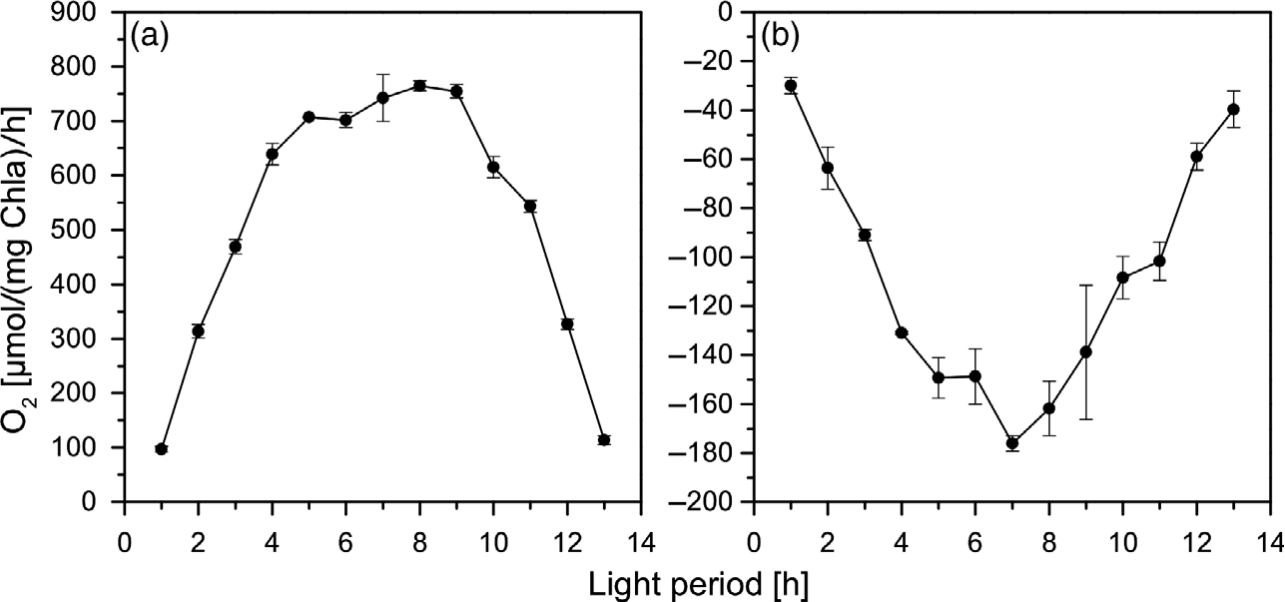
Gross oxygen production rates (a) and consumption via respiration (b) during a 14‐h illumination phase under biomass production conditions.

From Figure 2, it is evident that the photosynthesis and respiration rates under these conditions were strongly light‐ and temperature‐dependent. Accordingly, the gross oxygen production reached a maximum value of up to 800 μmol O_2_/(mg Chl*a*)/h at the peak of irradiance and temperature. This was accompanied by a strong increase of respiration rates in correlation with changes in irradiance and temperature. The obtained photosynthesis and respiration rates are in accordance with data of the studies of Torzillo *et al*. (2009) and Yang and Gao (2003) that were obtained under high CO_2_ supplement during cultivation. Obviously, *C. reinhardtii* was able to perfectly acclimate to these conditions and the cells did not show any signs of photoinhibition during maximum irradiance.

### Transition phase from biomass to glycolate production

**Figure 3:**
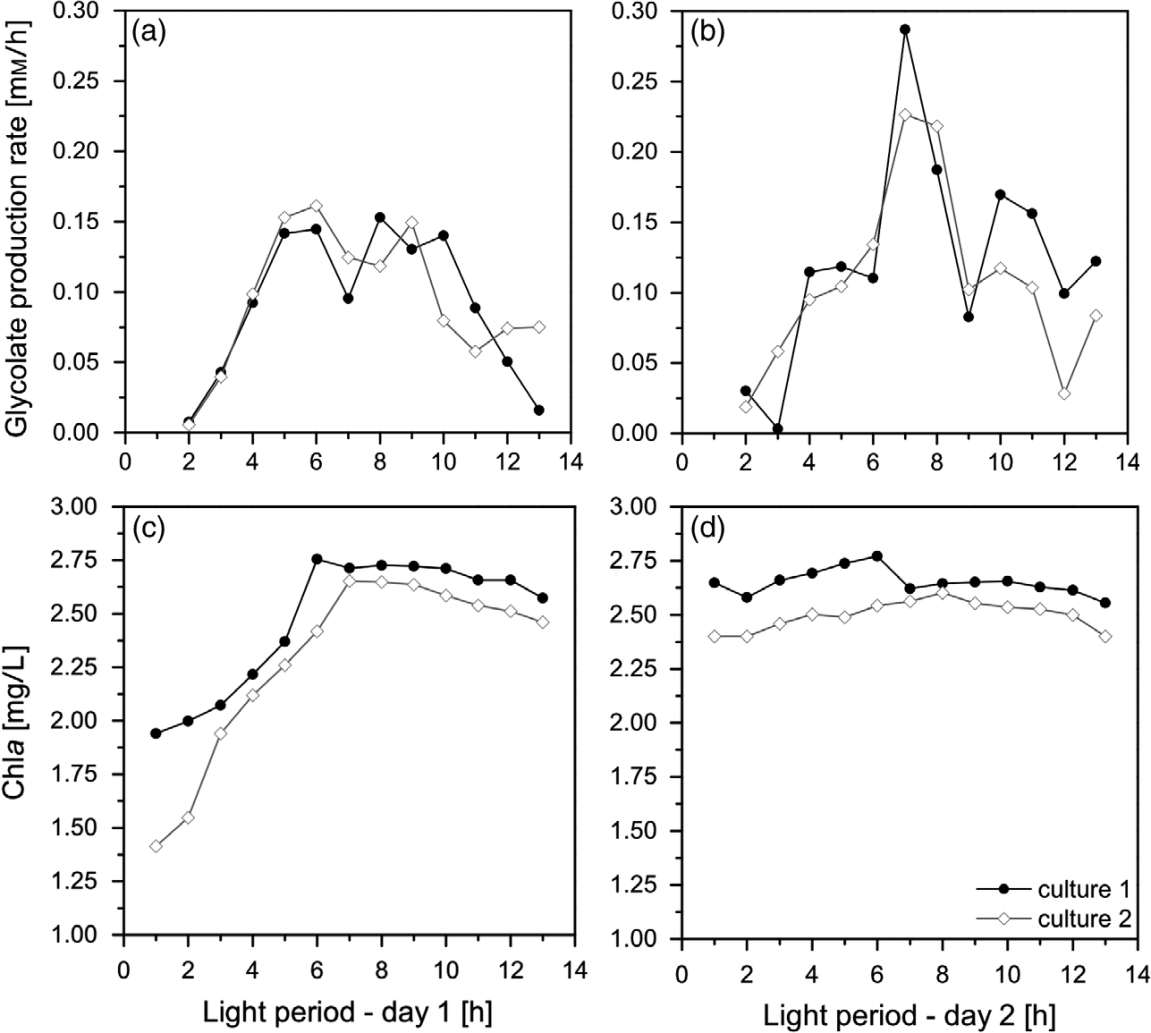
Glycolate production rates (a, b) and Chla concentration (c, d) of Chlamydomonas reinhardtii cultures during the first (a, c) and the second day (b, d) under photorespiratory conditions. Data were obtained from two independent algal cultures (Culture 1, closed circles; Culture 2, open diamonds).

**Figure 4:**
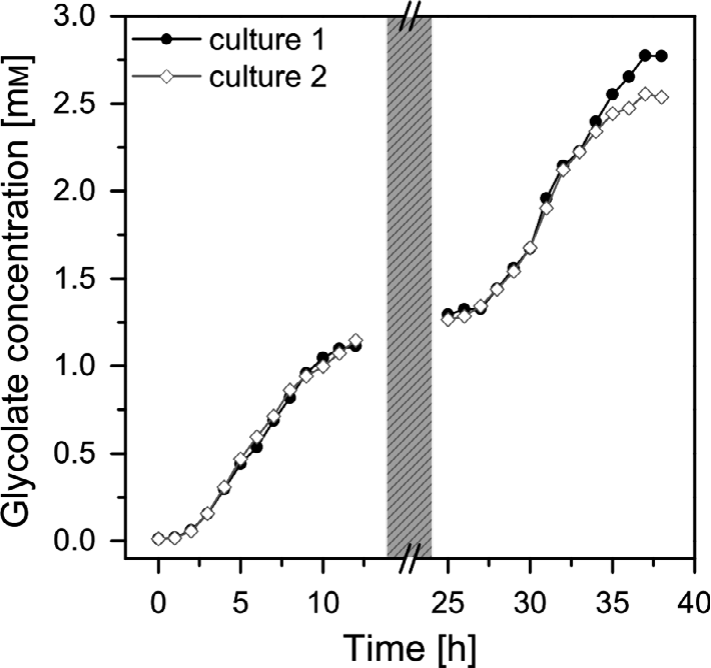
Glycolate accumulation [mM] in the medium of two independent algal cultures (Culture 1, closed circles; Culture 2, open diamonds) during the initial two illumination periods under photorespiratory conditions. Greyshaded area represents the dark phase between the illumination periods.

The cultures were forced into the glycolate‐producing phase by the change of aeration from 5% CO_2_ to a mixture of 40% O_2_/0.2% CO_2_ and by the addition of the inhibitor EZA. Figure 3a shows that at the first day the glycolate production follows the photosynthesis rates; however, there was a stagnation of glycolate production rates during midday. This stagnation disappeared from day 2 on under glycolate‐producing conditions (Figure 3b). It should be emphasized that this initial glycolate production rate was already three times higher than previously reported (Günther *et al*., 2012). The glycolate concentration reached a value of 1.25 mm on the first day and up to 2.75 mm on the second day (Figure 4). It has to be highlighted that there was no degradation of glycolate in the culture medium during the night phase. This excludes a re‐uptake of glycolate by the algal cells or a degradation of glycolate in the medium.

It is important to note that in parallel to the induction of glycolate excretion the cell growth stopped completely within 24 h. This is deduced from the changes of the Chl*a* concentration of the culture suspension (Figure 3c,d). Although the Chl*a* concentration significantly increased during the first day under glycolate‐producing condition, it remained constant from day 2 on. A similar effect was observed by the increase of cell numbers in parallel to Chl*a* concentration (data not shown). This means that the O_2_/CO_2_ ratio can be adjusted in a way that after an acclimation period of the cells any assimilated carbon was excreted as glycolate instead of biomass formation. As a practical consequence, the cultures were not diluted with fresh medium anymore to avoid a decline of biomass in the photobioreactors.

**Figure 5:**
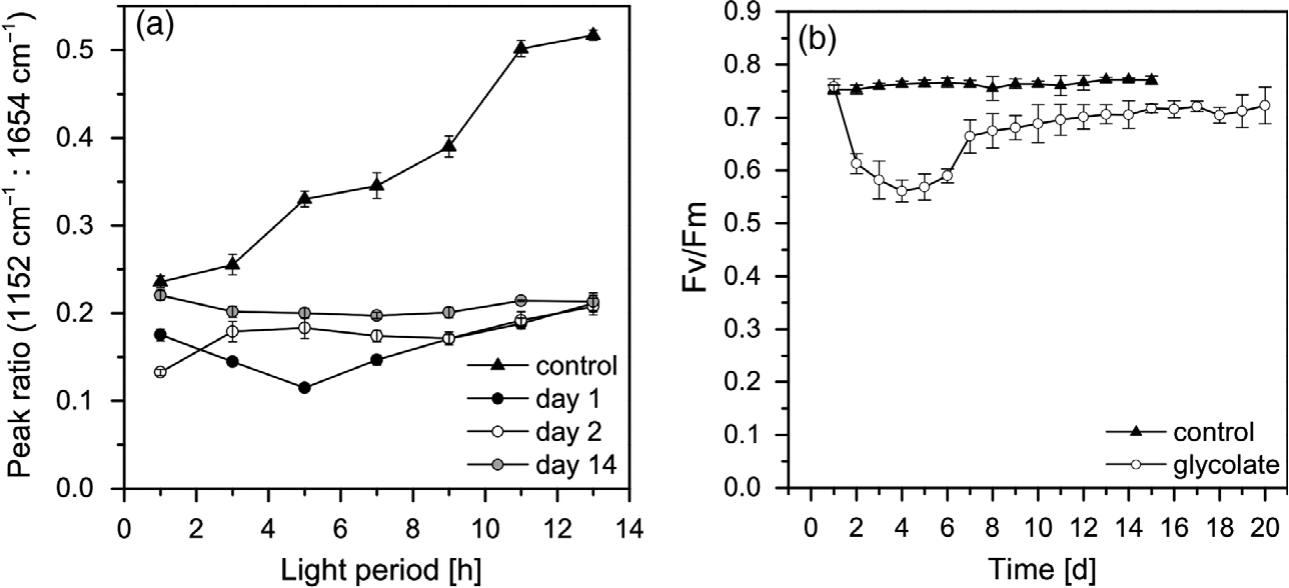
(a) Peak ratios of the carbohydrate vibration band (1152 cm^−1^) to the protein associated amide I vibration band (1654 cm^−1^) derived from FTIR spectra. Measurements were performed every 2 h during the 14‐h illumination period on cell samples under biomass production condition (closed triangles), at day 1 (closed circles), day 2 (open circles) and day 14 (grey circles) under glycolate production condition. (b) Maximum quantum efficiency of Photosystem II (Fv/Fm) of dark adapted cells for biomass‐(BM; closed triangles) and glycolate‐producing conditions (open circles).

During the shift from biomass‐producing to glycolate‐producing conditions, the cells were subject to severe changes in their metabolism. This was deduced from the changes in the macromolecular pattern measured by FTIR (Figure 5a; Figure S1). Under biomass‐producing conditions, carbohydrates accumulated in the light and were transferred to the other macromolecular pools (proteins and lipids) during the night. Such diurnal changes in macromolecular composition are typical for *Chlamydomonas* cells under light/dark cycle cultivation (Langner *et al*., 2009). In contrast, under the initial glycolate‐producing conditions the macromolecular composition did not change in course of the illumination period. This was most probably due to the fact that the assimilated carbon was preferentially channelled into the glycolate excretion pathway instead of biomass formation.

From these results it could be concluded that glycolate‐producing conditions induced a new physiological cell state that needs a molecular acclimation process. This assumption is supported by the severe decrease of the maximum quantum efficiency at PSII (Fv/Fm) from a mean value of 0.76 under biomass‐producing conditions to 0.61 and 0.58 on the first and second day under glycolate‐producing conditions, respectively (Figure 5b). A comparable observation was found in, e.g. maize leaves with an interrupted photorespiratory cycle and in the presence of glycolate or glyoxylate (González‐Moro *et al*., 2003). Since the glycolate excretion in *Chlamydomonas* was strongly enhanced under EZA treatment, it has to be concluded that the photorespiratory metabolism of glycolate was completely blocked. Therefore, the decrease of the PSII quantum efficiency is an indication of an inhibitory accumulation of photorespiratory products in the cells of *Chlamydomonas*. Such an accumulation does also inhibit the activity of RubisCO (Zelitch *et al*., 2009) that would explain the stagnation of glycolate excretion in comparison to photosynthesis rates during midday (see above).

### Glycolate production in long‐term experiment

For a biotechnological application of glycolate‐excreting algae, it is essential that the glycolate production can be kept constantly high for a long time period without negative impact on photoassimilation. Thus, the investigation of the long‐term stability of glycolate excretion was a central question of the present study.

**Figure 6:**
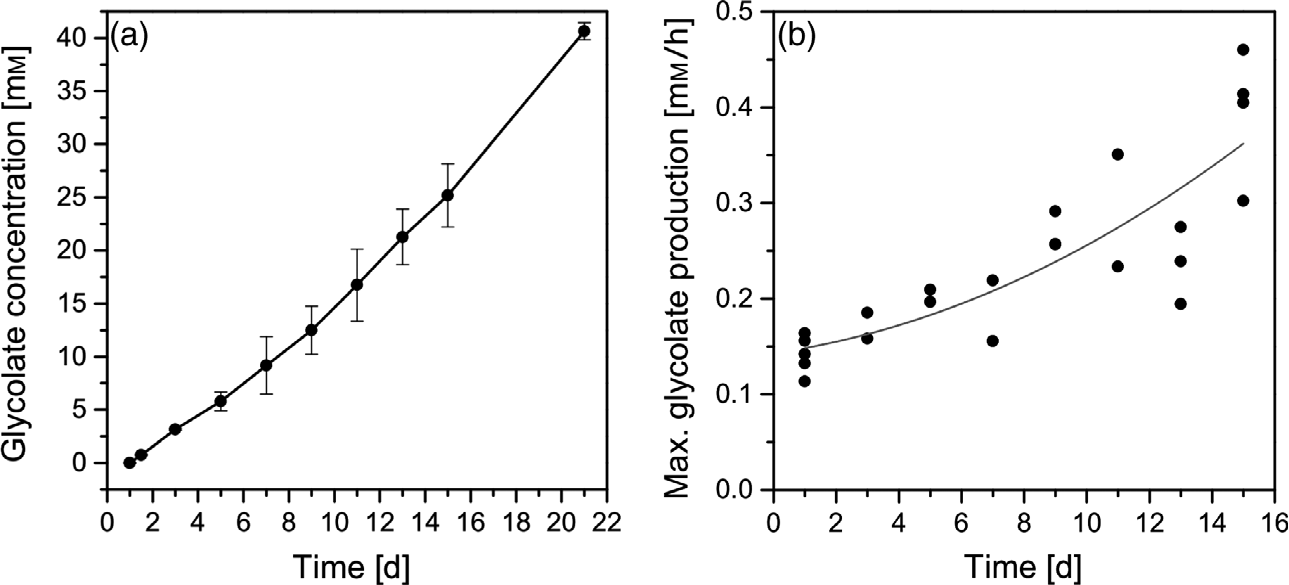
(a) Total glycolate accumulation [mm] in the culture medium under photorespiratory conditions within the experimental period of 20 days and (b) maximum glycolate production rates [mm/h] measured at the daily peak of irradiance and temperature within the experimental period of 14 days.

In the present study, the glycolate excretion was monitored over a period of up to 21 days. Figure 6a shows that glycolate accumulates in the medium up to a concentration of 41 mm within 21 days. The experiment was stopped at this time point due to the limited available volume of algal suspension for sampling. Importantly, the daily glycolate excretion rate did not only remained constant but tended to further increase over this 21‐day period. From Figure 6b, it could be deduced that particularly the maximum glycolate excretion rate at midday steadily increased during the entire experimental period. Accordingly, the maximum glycolate production rate increased from 0.13 mm/h at day 1 to about 0.35 mm/h at day 15. Obviously, *C. reinhardtii* cells did acclimate to the photorespiratory conditions and were able to perfectly balance the flux of light energy uptake to carbon flow. On the one hand, this is a surprising result since photosynthetic cells usually try to keep photorespiration at a minimum level (e.g. by activation of CCMs) to avoid the inevitable loss of assimilated carbon. On the other hand, in the presence of increased oxygen concentrations the oxygenase activity of RubisCO is inevitable and the avoidance of (toxic) glycolate accumulation by the photorespiratory cycle or by excretion of glycolate can be seen as the basic function of photorespiration (Zelitch *et al*., 2009) and it is essential to keep this function active even on long‐term periods. In addition, it should be highlighted that *C. reinhardtii* cells of the present study were in the unusual situation of high metabolic activity but channelled photosynthetic energy and assimilated carbon completely into the synthesis of glycolate instead of biomass production. Consequently, it has to be assumed that no assimilated carbon left the chloroplasts in form of triose phosphates and completely prevented cellular sugar synthesis. It is known that sugars (e.g. glucose, sucrose) and sugar accumulation are developmental triggers and are involved in the regulation of plant growth. Particularly, the hexokinase is an important sensing and signalling element (reviewed in Rolland *et al*., 2006). To our knowledge, the described effect of metabolically highly active but not growing cells was not observed before and could contribute to the interpretation of the function of photorespiration and to the regulation cell growth in unicellular algae (Davis *et al*., 2013).

The enhancement of glycolate production was accompanied by a steady increase of the quantum efficiency at PSII between day 2 and day 15 (Figure 5b). Thus, the cells were obviously able to balance the flux of light energy uptake to carbon flow under these conditions. It also means that the increasing concentration of glycolate in the surrounding medium and the constant presence of the inhibitor EZA did not negatively affected the fitness of the cells. It should be also noted that EZA was obviously not degraded during the glycolate‐producing condition which is an important aspect for a biotechnological application. It should be noted that Fv/Fm values under glycolate‐producing conditions remained always slightly lower than the values under biomass‐producing conditions. It could be assumed that this observation is due to a general inhibitory effect of photorespiration either directly on the quantum efficiency and electron transport out of PSII (Messant *et al*., 2018) or on RubisCO activity (González‐Moro *et al*., 1997).

### Biotechnological applicability

Several aspects have to be considered to qualify glycolate‐producing algae for a biotechnological application in a continuous production process. These include a stable physiological cell state (ideally homoeostasis), a high efficiency of glycolate production from assimilated carbon, and a sufficient product titre to avoid additional concentration steps.

The FTIR spectra measured on day 2, day 8 (data not shown) and day 15 (Figure 5a, Figure S1) revealed only slight changes of the macromolecular cell composition in course of the daily illumination period. Such a stable macromolecular composition is a typical characteristics of metabolically homoeostatic cells (Wagner *et al*., 2014). Thus, even under long‐term photorespiratory conditions, the cells were in a balanced state and the allocation of assimilated carbon which was not excreted as glycolate was optimized to remain the physiological activity of the cells irrespectively of the growth cessation. This is in line with the observed recovery of the activity of the photosynthetic apparatus under glycolate‐producing conditions (see above). Such a homoeostatic metabolic cell status is a robust condition for biotechnological practice and a continuous production process.

The efficiency of the glycolate excretion was evaluated by comparing the theoretically expected with the practically achieved glycolate production rate. The expected glycolate production rate was estimated from the carbon assimilation rate under biomass production condition with the assumption that it represents the carboxylation, and thus, the total activity of RubisCO. It is further considered that the biosynthesis of glycolate requires one oxygenation reaction by RubisCO but two additional carboxylation reactions to re‐assimilate two molecules of carbon that are excreted as glycolate. Accordingly, from the measured carbon assimilation rate under biomass‐producing condition (Table 1), an expected glycolate‐based carbon excretion of 2.33 mmol C/(mg Chl*a*)/day (equals 1.17 mmol glycolate/(mg Chl*a*)/day) was estimated. The comparison of this expected values to the achieved glycolate excretion revealed a strong increase of the efficiency of the process from 36% during the initial phase to 82% at the end of the experimental period under glycolate‐producing conditions (after 3 weeks; Table 1).

The high efficiency of glycolate production was accompanied by the accumulation of glycolate in the surrounding medium up to a concentration of 3.1 g/L. This is significantly higher than the glycolate concentration that was evaluated as the maximum loading of a glycolate fermenting biogas reactor (1.8 g/L; Günther *et al*., 2018). Based on these results, a further energy‐consuming product concentration is not necessary. Together with the high efficiency of glycolate production and the estimated production potential (see below), it strongly promotes the glycolate‐based energy and CO_2_ conversion technology.

### Estimation of the potential of glycolate‐based methane production

In Günther *et al*. (2012), it was shown that methane can be produced from glycolate with a yield of 0.173 m^3^/kg. Based on the measured maximum glycolate production rate of the present study (Table 1) and further assumptions (see Experimental procedures section), the potential of methane production can be estimated and represents the current potential of glycolate‐based methane production. Accordingly, a glycolate production rate of about 800 g/m^2^/a and a resulting methane production rate of about 1400 m^3^/ha/a was estimated. This is equivalent to an energy yield of 50 GJ per hectare and year.

In the present study, the photobioreactor was operated at relatively low cell concentration to allow for a reliable online measurement of cell density and variable Chl fluorescence and to keep the cell cultures in a homoeostatic cell state. However, photobioreactors are typically operated at high Chl concentration to enhance their area‐based productivity. Based on the absorption properties of the cells of *C. reinhardtii*, a biomass concentration of 300 mg Chl per m² corresponds to an absorption efficiency of incident irradiance of about 90%. On the basis of the measured mean glycolate production rate of 60 μmol/(mg Chl)/h, the estimated methane production increases to 5300 m³/ha/a and is equivalent to an energy yield of 191 GJ per hectare and year. Even in consideration of energy losses for the operation of a proposed biofilm‐based photobioreactor and for biogas production (28% of gross energy production; Weinberg *et al*., 2012), the estimated net energy yield of 138 GJ/ha/a is significantly higher than the average energy yield of 90–100 GJ/ha/a of the most efficient conventional energy plant maize (Felten *et al*., 2013). In the light of the ‘food versus fuel’ debate, the energy production by employing algal biomass cultivated in photobioreactors possesses an intrinsic advantage. However, due to the very high energy input requirement for, e.g. nutrient provision and harvesting of algal biomass, there is usually no positive net energy gain obtained from the fermentation of algal biomass or the production of biodiesel from algal oil (Jorquera *et al*., 2010; Razon and Tan, 2011). By contrast, in the glycolate‐based approach the situation changes completely because nutrient provision can be drastically reduced, the harvesting of biomass is completely avoided and thus, a positive energy gain be achieved.

## Conclusions

In the present study, the green alga *C. reinhardtii* was cultivated continuously in a photobioreactor under glycolate‐producing conditions. The experimental results allow to answer basic questions as prerequisites for a biotechnological application of this approach. (i) The high photosynthetic activity of the cells resulted in a high and long‐term stable glycolate production rate. The algal cells proved to be tolerant against the permanent photorespiratory burden and the high glycolate accumulation in the culture medium at the end of the experimental period. (ii) The achieved glycolate concentrations allow a direct usage for fermentation processes without any further steps of concentration. (iii) The O_2_/CO_2_ ratio can be adjusted in a way that the assimilated carbon is excreted completely as glycolate. The resulting growth cessation opens the possibility to use an algae‐biofilm technology instead of suspension cultures that would strongly increase the energetic efficiency of the production system. Thus, algal‐based glycolate excretion can be run as a continuous process where no harvest of algal biomass is necessary. (iv) Algal‐based glycolate excretion proved to be a robust process even under conditions with oscillating light and temperature conditions.

## Experimental procedures

### Culture conditions

The green alga *Chlamydomonas reinhardtii* (SAG 11‐32b, Culture Collection of Algae, Göttingen, Germany) was cultivated in a batch preculture at 20 °C and at a light intensity of 100 μmol photons/m^2^/s white light (L36W/840, Osram, Munich, Germany) with a light/dark cycle of 14/10 h. A modified Tris‐Phosphate minimal medium (TP) (Gorman and Levine, 1965) was utilized with double Tris buffer (39.95 mm), the addition of 3.08 μm FeSO_4_ × 7H_2_O plus 2.3 μm Na_2_‐EDTA and the use of the trace metals solution of the Bold's Basal medium (Bischoff and Bold, 1963) instead of the Hunter trace element solution. The pH of the medium was adjusted to a value of 7.0.

For the experiments, the cells were grown continuously in a flat 400 mL photobioreactor (FMT 150, PSI, Drasov, Czech Republic) with an illuminated area of 0.016 m², in the modified TP medium and aerated with CO_2_‐enriched air (21% O_2_/5% CO_2_). Temperature gradients (15–30 °C) and dynamic light conditions (1700 μmol photons/m/s maximum irradiance) were applied to simulate outdoor conditions of a summer day in the temperate zone (14/10 h light/dark) (Figure 1).

Algal cultures were kept at a chlorophyll *a* (Chl*a*) concentration between 2 and 3 mg Chl*a*/L by continuous dilution with fresh medium. The dilution with medium was controlled by an online measurement of the optical density (OD) at 680 nm. Physiological measurements were started after 1 week of stable growth.

Daily growth rates (μ) were calculated according to the following equations:

(1) $$\mu \;\left(d^{-1}\right)=D+\left(\frac{dx}{dt}\times x_{0}\right)$$

(2) D=f/V

where *D* is the daily dilution rate, *f* is the dilution volume, *V* is the volume of the culture vessel and d*x* is the change in Chl*a* concentration of time interval d*t*.

### Determination of cell dry weight, cell number and chlorophyll content

For the determination of cell dry weight 25 mL of the algae cultures were harvested by centrifugation (2500×*g*, 10 min, 5°C; Sigma 2‐16k; Sigma, Osterode am Harz, Germany). After resuspending and washing the cells twice with 25 mL deionized water, cells were taken up in 1.5 mL of deionized water and freeze‐dried (Labconco Freezone 2,5; Ilmvac GmbH, Ilmenau, Germany). The cell number was counted by means of a cell counter (Z2 Coulter Counter, Coulter Electronics Inc., Miami, FL). Samples for measurements of Chl content were taken at the beginning of the light period and at the time point of maximum irradiance and temperature. Determination of Chl concentration was carried out spectrophotometrically corresponding to Ziegler and Egle (1965). Therefore, 5–10 mL algae suspension was harvested on a glass fibre filter (MN 85/70 Ø 25 mm, Macherey‐Nagel, Dueren, Germany). After addition of 2.5 mL of 80% acetone and 2.5 g glass beads (∅ 0.25–0.3/1.00–1.05 mm; 3:1) cells were broken in a cell homogenizer (20 s at 6500 rpm; Precellys Evolution, Bertin Technologies, Paris, France). The pigment extract was centrifuged for 2.5 min at 16 000×*g* (Sigma 1‐14; Sigma) and the absorption was measured at the wavelengths 664 and 647 nm (U2000 spectrophotometer, Hitachi, Tokyo, Japan).

### Determination of oxygen evolution and variable chlorophyll fluorescence

The oxygen evolution rates and the fluorescence parameters were measured simultaneously by means of a combination of pulse amplitude modulation (PAM) fluorometer (unit 101/103, Walz, Effeltrich, Germany) and light pipette (Illuminova, Uppsala, Sweden) equipped with a special cuvette (Topgallant LLC, Salt Lake City, UT) connecting the emitter and the detector unit of the PAM fluorometer. The light pipette was used as actinic light source (Xenophot Longlife HLX64642; Osram, Munich, Germany) and for the oxygen evolution measurements using a Clark‐type electrode (MI 730; Microelectrodes Inc., Bedford, New Hampshire, MA). During the light phase, every hour 3 mL of the algal suspension was taken out of the photobioreactor and placed in the cuvette of the device. Net oxygen evolution rates [μmol O_2_/(mg Chl*a*)/h] were measured for 10 min at five different actinic light intensities (91, 341, 691, 1064, 1386 μmol photons/m^2^/s) and temperatures (15, 17, 20, 24, 27, 30 °C) to take into account the daily course of irradiance and temperature in the photobioreactor. Respiration rates [μmol O_2_/(mg Chl*a*)/h] were measured after the respective illumination for 10 min in darkness. Gross photosynthesis rate was derived by correcting the net oxygen evolution rates for the corresponding dark respiration rate.

The maximum quantum yield of Photosystem II (Fv/Fm) was measured directly on the cultures in the photobioreactor at the end of the dark period just before the start of daily light phase (PSI, Drasov, Czech Republic). The fluorescence method's principles are based on Schreiber *et al*. (1995). The online fluorescence data were used to estimate the physiological state of the cultures under glycolate‐producing conditions.

### Calculation of primary production

For the estimation of the primary production, the measured values of the dry weight (DW) [mg/(mg Chl*a*)] and the growth rate (μ)/day were used based on equation (3):

(3) $$C_{\mathrm{BM}}=\frac{\text{DW}\;\times \;\mu }{m_{C}\;\times \;f_{c}}$$

where *m*_*C*_ is the molecular weight of carbon (12.01 g/mol) and *f*_*c*_ is the proportion of carbon per dry mass. According to Langner *et al*. (2009) *f*_*c*_ is 2 for *C. reinhardtii*.

### Experimental setup for glycolate production and determination of glycolate excretion rates

For glycolate production/excretion, the aeration of algal cultures was changed from biomass‐producing conditions (21% O_2_/5% CO_2_) to photorespiratory conditions with 40% O_2_ and 0.2% CO_2_. The inhibitor EZA (6‐Ethoxy‐2‐benzothiazolesulfonamide; Sigma‐Aldrich, Darmstadt, Germany) was added at a concentration of 0.05 mm to prevent the induction of carbon‐concentrating mechanisms under photorespiratory conditions. For determination of glycolate concentration in the medium of algal cultures, samples (2 mL) were collected every hour during illumination phase the first 2 days and later every second day 1 h before and after light maximum, sterile filtered (PES 0.22 μm; Carl Roth, Karlsruhe, Germany) and stored at −20 °C until further use.

For the quantitative determination of glycolate concentration, the colorimetric method with 2.7‐dihydroxynaphthalene (Takahashi, 1972) was used. Accordingly, 50 μL of the sample was dissolved in 1.5 mL of the staining reagent and incubated in a water bath at 100 °C for 20 min. The reaction was stopped on ice and the absorbance of the samples was measured at 540 nm (Specord M250, Zeiss, Jena, Germany).

### Comparison of carbon allocation via FTIR spectroscopy

For the assessment of the carbon allocation during the different conditions a bench‐top Fourier‐transform infrared spectroscopy (FTIR) analysis was carried out as described by Fanesi *et al*. (2017). Biomass and glycolate production conditions samples were taken every second hour during the light phase. Cells were harvested by centrifugation of 1.5 mL algal suspension for 2 min at 2500×*g* (Minispin, Eppendorf, Hamburg, Germany). The pellet was resuspended two times in 1 ml distilled water to remove cell fragments as well as salt residues and centrifuged again. Depending on the final cell concentration the pellet was resuspended in 25–90 μL distilled water, to reach a final cell concentration in order to record spectra with a maximum absorption between 0.15 and 0.3 absorbance units. 2 μL of the concentrated cell suspension was placed on a silicon 384 well‐microplate (Bruker Optics, Ettlingen, Germany) and dried at 40 °C for at least 30 min (cabinet dryer, Thermo Fisher Scientific, Hanau, Germany). Recording of the spectra was carried out in transmission mode with 32 scans. In the spectral range of 4000–700 cm^−1^, spectra were co‐added and averaged. Measurements were computer controlled (OpusLab v. 5.0 software, Bruker Optics, Ettlingen, Germany) and carried out with a Bruker Vector 22 spectrometer connected to a HTS‐XT microplate reader (Bruker Optics, Ettlingen, Germany). Recorded spectra were baseline corrected by the rubber band algorithm (OPUS v. 5.0 software). The peak ratio of the carbohydrate‐associated vibration band (1152 cm^−1^) and the amide I (protein) vibration band (1654 cm^−1^) was calculated. Five technical replicates of two biological samples were measured and finally normalized to amide I vibration band (1654 cm^−1^) for comparative presentation.

### Estimation of glycolate‐based methane production

To demonstrate the biotechnological potential of the presented approach, the expected glycolate‐based methane production was estimated. The glycolate‐based methane production was estimated for two different scenarios: (i) an upscaling of the present photobioreactor setup with a Chl*a* content between 2 and 2.8 mg/L (equals 50–70 mg Chl*a*/m^2^) and an illuminated area of 0.04 m^2^/L algal suspension, and b) a hypothetical photobioreactor setup with a high Chl concentration of up to 300 mg Chl*a*/m^2^. In the latter scenario, it is assumed that the same glycolate production rate [e.g. mmol glycolate/(mg Chl*a*)/h] is achieved as in the current photobioreactor setup. Further, a ‘vegetation period’ from April to September with T > 10 °C is assumed. For this period, a total of 2240 h of solar insolation is derived for a location in central Germany on the basis of the daily solar insolation (minus 2 h for sunrise and sunset). With these assumptions, the area‐based glycolate production was estimated and converted into the expected amount of methane by the measured factor of 0.173 L per kg glycolate (Günther *et al*., 2012). From methane production, the expected energy yield was calculated with the assumption of 36 MJ per m³ methane.

## Competing interests

The authors declare no competing financial interests.

## Supporting information


**Figure S1** FTIR spectra of *C. reinhardtii* at biomass production, 1st, 2nd and 15th day of glycolate production during sine phase.
