# Supplementary material for: Glycolate from microalgae: an efficient carbon source for biotechnological applications
Source: Plant Biotechnol J. 2019 Feb 4;17(8):1538–46. doi: 10.1111/pbi.13078 (PMC6662103; doi:10.1111/pbi.13078)
Supplement: Supplementary file 1 — Figure S1 FTIR spectra of C. reinhardtii at biomass production, 1st, 2nd and 15th day of glycolate production during sine phase. [file PBI-17-1538-s001.docx]

**Supplement**

**Supplemental Figure 1**: FTIR-Spectra of *C. reinhardtii* at biomass production, 1st, 2nd, 15th day of glycolate production during sine phase. Spectra were normalized to the amide I vibration band (1654 cm^-1^) and plotted as relative absorbance units.
